# Physiological and Molecular Responses of Seed Germination to Irrigating-Sowing in Drought-Stressed Foxtail Millet (*Setaria italica* L.)

**DOI:** 10.3390/plants14233571

**Published:** 2025-11-22

**Authors:** Boyu Lu, Shide Dan, Siyu Yan, Rongxue Wang, Jiaxing Li, Jianhong Ren, Shuqi Dong, Yinyuan Wen, Liguang Zhang, Xiangyang Yuan

**Affiliations:** 1 College of Agriculture, Shanxi Agricultural University, Jinzhong 030801, China; 18735424089@163.com (B.L.);; 2 College of Life Sciences, Shanxi Agricultural University, Jinzhong 030801, China; 3 Special Orphan Crops Research Center of the Loess Plateau, MARA, Shanxi Agricultural University, Jinzhong 030801, China

**Keywords:** foxtail millet, drought, irrigating-sowing, seed germination, transcriptome analysis, carbohydrate metabolism, secondary metabolism, hormone signaling

## Abstract

Foxtail millet (*Setaria italica* L.) is an important crop in northern China’s arid and semi-arid regions. Frequent spring droughts and limited irrigation facilities often cause poor seed germination due to insufficient soil moisture, threatening food security. The irrigation-sowing technique, which creates a localized moist microenvironment around seeds, effectively addresses this issue. However, this technique has been poorly studied, and its effects on foxtail millet seed germination remain unclear. To address this, field experiments were conducted using a two-factor split-plot design, with three drought levels and five irrigation gradients. The results showed that irrigation-sowing increased soil moisture, promoted root–shoot growth coordination, and improved germination characteristics. Transcriptome analysis of seeds under moderate drought compared the optimal irrigation treatment (13.5 m^3^·hm^−2^) with the non-irrigated control (0 m^3^·hm^−2^), identifying 2169 differentially expressed genes. Seeds receiving irrigation exhibited higher transcript abundance in pathways related to carbohydrate metabolism, energy production, secondary metabolism, and hormone signaling. Physiological measurements further showed increased α/β-amylase activity, while starch, sucrose, and cellulose content decreased. Glycolytic enzyme activity was enhanced, and ATP content increased by 125%. Additionally, phenylpropanoid metabolism was promoted, and proanthocyanidin accumulation increased by 11.5%. Hormone analysis showed that the contents of IAA and GA increased as germination progressed by 29.09% and 54.70%, respectively, while ABA content decreased. Overall, irrigation-sowing serves as an upstream moisture signal that reshapes metabolic and hormonal states associated with improved germination performance.

## 1. Introduction

Foxtail millet (*Setaria italica* L.) is an important crop in the arid and semi-arid regions of northern China, with a cultivation history of approximately 8700 years [1]. Its grains and stems serve both as food and fodder, making it an essential crop in dryland agriculture in northern China [2,3]. Foxtail millet primarily grows in arid regions such as the Loess Plateau and hilly areas, where natural precipitation and ample sunlight align well with its drought-tolerant and light-demanding growth characteristics. However, with the intensification of climate change and the increasing issue of water scarcity, frequent spring droughts in northern regions often result in insufficient soil moisture, causing low seedling emergence rate and threatening food security. At the same time, these regions lack irrigation infrastructure, and traditional surface or furrow irrigation is impractical due to the instability of water sources. Advanced systems like drip and sprinkler irrigation, due to their high cost and technical requirements, are difficult to implement in rural areas [4]. Although foxtail millet is a drought-tolerant cereal crop, its seed germination and seedling growth are highly sensitive to water availability, and water deficit often leads to poor germination and reduced seedling vigor [5]. Studies show that the moisture content in the 0–20 cm topsoil is critical for foxtail millet’s germination and early growth. When soil moisture falls below the germination threshold, foxtail millet seeds cannot absorb enough water, leading to delayed or failed germination [6]. Even if some seeds germinate, the rapid depletion of soil moisture can cause seedling desiccation, resulting in sparse and uneven emergence in the field, ultimately leading to insufficient seedling populations and severe yield reductions. Therefore, improving seed germination and seedling vigor is crucial for increasing yield. Once seedlings emerge, foxtail millet can usually rely on natural rainfall for the subsequent growth and yield formation.

To address this challenge, farmers in China have developed irrigation-sowing, a technique that involves applying water around the seeds during sowing to create a localized moist environment that meets the water requirements for early germination, ensuring full emergence [7,8,9,10]. Unlike traditional large-scale irrigation methods, irrigation-sowing focuses on precision irrigation, preventing water wastage and offering a high-efficiency, water-saving alternative [4]. The 2024 Corn Yield Increase Manual by the Ministry of Agriculture and Rural Affairs of the People’s Republic of China that irrigation-sowing in Northeast China increases seedling emergence by more than 30% [11], significantly improving emergence uniformity and yield. However, while the technique has shown notable success in other crops like maize [7,8,9,10], its effects on foxtail millet seed germination, especially at the physiological and molecular levels, remain poorly understood.

Water is essential for seed germination, which involves sequential physiological stages of water uptake, metabolic activation, and mobilization of stored reserves for energy production [6,12]. Drought disrupts this process by reducing seed water absorption, leading to lower germination rate and seedling vigor [13]. Drought stress delays water uptake and chromatin activation, which consequently reduces amylase activity and slows starch hydrolysis into soluble sugars, thereby limiting energy availability during germination [12,13,14,15,16,17]. At the metabolic level, drought disturbs the coordination among glycolysis (EMP), the tricarboxylic acid cycle (TCA), and the pentose phosphate pathway (PPP), reducing ATP and NADPH generation and ultimately weakening energy metabolism [18,19]. Meanwhile, the activation of the phenylpropanoid pathway under drought leads to the accumulation of flavonoids, anthocyanins, and lignins that strengthen cell walls and enhance antioxidant capacity [20,21,22], but excessive deposition may hinder cell expansion and radicle emergence.

Hormonal regulation further modulates these drought responses. Drought elevates abscisic acid (ABA) and suppresses gibberellin (GA) synthesis, increasing the ABA/GA ratio and inhibiting germination through the ABI5 and DELLA pathways [23,24,25]. Ethylene can alleviate ABA-induced inhibition by promoting seed coat rupture [26,27], whereas jasmonic acid (JA) and auxin (IAA) can cooperate with ABA under stress conditions such as drought to enhance dormancy maintenance via the ARF10/ARF16–JAZ–ABI5 regulatory module, while the oxylipin OPDA also acts synergistically with ABA to inhibit germination [28,29,30]. In contrast, brassinosteroids (BRs) and cytokinins (CTKs) cooperate with GA to stimulate energy metabolism, cell division, and elongation during seed germination and early seedling growth, although their levels decline under drought stress [31,32,33,34]. Collectively, drought exerts a multi-level inhibitory effect on seed germination by impairing water uptake, disturbing energy and secondary metabolism, and reshaping hormonal homeostasis.

In recent years, field-scale transcriptomics has attracted increasing attention for its ability to elucidate crop stress-response mechanisms under real agricultural conditions. RNA-Seq technology has been successfully applied to field-based stress studies in crops such as maize and sorghum [35], while foxtail millet, with its small genome, low repetitive sequences, and well-annotated genomic information, exhibits unique potential in this field [36,37]. Although numerous drought-responsive genes and pathways have been reported, the enzymes and transcription factors acting downstream of these genes also play indispensable roles in various biological processes. To cope with drought stress, plants have evolved complex adaptive strategies, including metabolic remodeling, epigenetic plasticity, and gene expression reprogramming [38]. Against this background, the integration of field-scale transcriptomic and physiological analyses provides an effective approach to characterize the coordinated physiological and molecular responses of foxtail millet seeds to irrigating-sowing during germination, thereby offering new insights into the basis of drought-resilient seedling establishment in foxtail millet.

This study uses Jingu 21 as the research material, as it is a representative variety of high-quality foxtail millet in China, and the perennial sowing area is large. While irrigation-sowing has shown significant contributions to water conservation and seedling protection in other crops like maize, systematic research on its effects on small-grain drought-tolerant crops like foxtail millet is limited, particularly regarding its physiological and molecular impacts on early germination. The seed germination process begins with water uptake, followed by chromatin remodeling that activates transcription and metabolism for energy production [14,15,16]. These molecular and physiological changes are accompanied by the re-establishment of auxin gradients and the spatial activation of metabolism across different cell types and positions within the embryo [12,13,14,15,16,17]. Considering the important roles of carbohydrate mobilization, energy metabolism, secondary metabolite synthesis, and hormone balance in seed germination, we hypothesize that irrigating-sowing may generate more favorable states of carbohydrate mobilization, energy metabolism, secondary metabolite synthesis and hormone balance that are associated with greater germination vigor of foxtail millet seeds. Accordingly, this study aimed to investigate how irrigating-sowing influences carbohydrate mobilization, energy metabolism, secondary metabolite synthesis and hormone-related traits during millet seed germination.

## 2. Results

### 2.1. Effect of Irrigating-Sowing on Seed Germination Characteristics in Foxtail Millet

**Figure 1:**
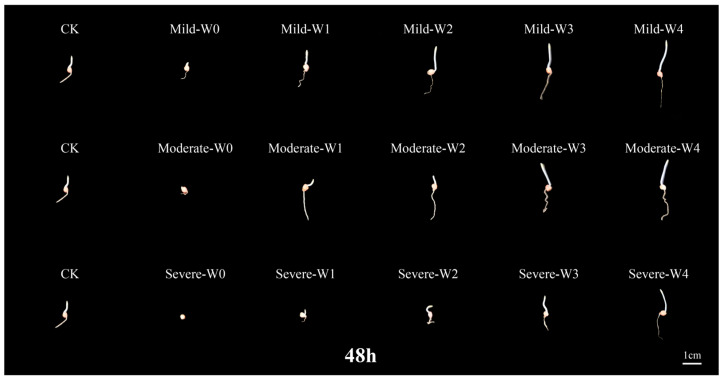
Differences in germination morphology of foxtail millet seeds under irrigating-sowing. Drought levels: mild (60% ± 5%), moderate (50% ± 5%), and severe (40% ± 5%) field water holding capacity. Irrigation treatments: W0 (0 m^3^·hm^−2^), W1 (4.5 m^3^·hm^−2^), W2 (9 m^3^·hm^−2^), W3 (13.5 m^3^·hm^−2^), and W4 (18 m^3^·hm^−2^). CK represents the normal field capacity (70% ± 5%). Seeds were photographed 48 h after sowing, and the scale bar indicates 1 cm.

In the 48 h germination period of foxtail millet seeds, seed germination was substantially inhibited as drought severity increased, with inadequate root and shoot development. Under three different drought conditions, increase irrigation improved the seedling growth characteristics, indicating that appropriate irrigation can effectively alleviate the adverse effects of drought stress on seed germination (Figure 1).

**Figure 2:**
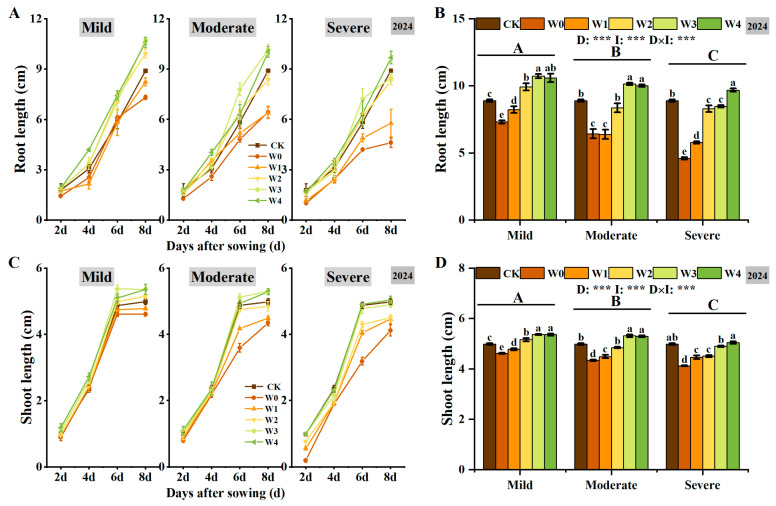
Effects of irrigating-sowing on root and shoot growth of foxtail millet. (**A**,**C**) Changes in root (**A**) and shoot (**C**) length of foxtail millet seedlings at 2, 4, 6, and 8 days after sowing under three drought levels (mild, moderate, and severe) with five irrigation treatments (W0–W4). CK represents the well-watered control (70% ± 5% of field capacity). (**B**,**D**) Comparison of root (**B**) and shoot (**D**) length on the 8th day under different drought and irrigation treatments. Error bars represent the standard error of the mean (*n* = 3). Different uppercase letters indicate significant differences among drought levels (*p* < 0.05), and different lowercase letters indicate significant differences among irrigation treatments within the same drought level (*p* < 0.05). D, I, and D × I represent the main effects of drought, irrigation, and their interaction, respectively. *, **, and *** indicate significant differences at *p* < 0.05, *p* < 0.01, and *p* < 0.001, respectively.

Irrigated sowing significantly influenced root and shoot growth in foxtail millet, with continuous growth observed over time (Figure 2). Root growth was more pronounced than shoot growth (Figure 2A,C), indicating that foxtail millet seeds prioritize root development to enhance water acquisition. Under three different drought conditions, both root and shoot lengths increased with higher irrigation. Under moderate drought, by the 8th day, root length in the W3 increased by 57.74% compared to W0, and shoot length increased by 22.37% (Figure 2B,D). Additionally, higher shoot length was observed on the 6th day, with slower elongation thereafter (Figure 2C), indicating that irrigating-sowing helps promote early rapid shoot elongation.

**Figure 3:**
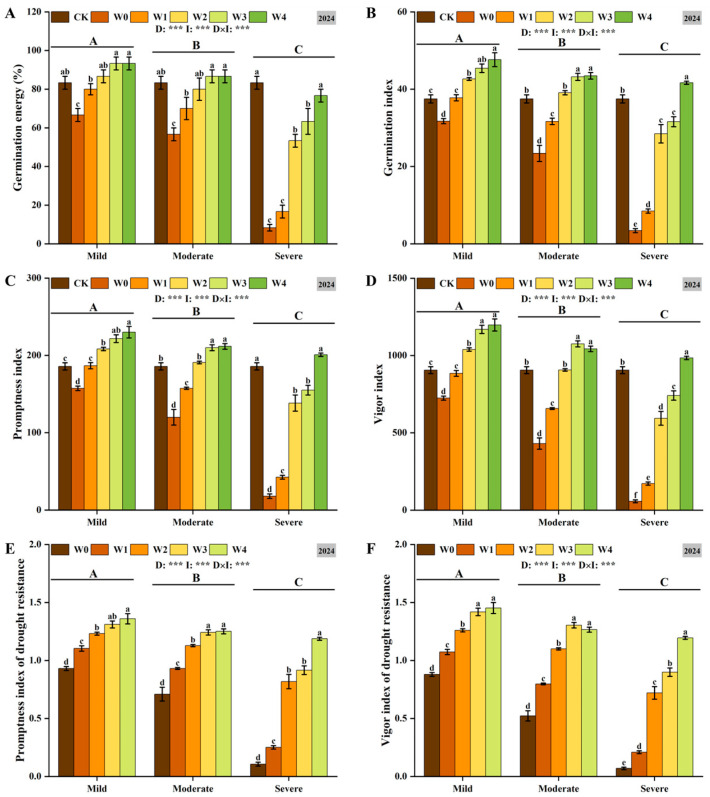
The changes in germination energy (**A**), germination index (**B**), promptness index (**C**), vigor index (**D**), promptness index of drought resistance (**E**) and vigor index of drought resistance (**F**) of foxtail millet seeds under irrigating-sowing. Error bars represent the standard error of the mean (*n* = 3). Different uppercase letters indicate significant differences among drought levels (*p* < 0.05), and different lowercase letters indicate significant differences among irrigation treatments within the same drought level (*p* < 0.05). D, I, and D × I represent the main effects of drought, irrigation, and their interaction, respectively. *, **, and *** indicate significant differences at *p* < 0.05, *p* < 0.01, and *p* < 0.001, respectively.

Drought stress significantly inhibited seed germination, and as drought severity increased, germination energy, germination index, promptness index, vigor index, drought resistance germination index, and drought resistance vigor index decreased significantly (Figure 3). Irrigating-sowing effectively alleviated the adverse effects of drought. Under mild and moderate drought conditions, both W3 and W4 significantly improved seed germination characteristics, with no significant difference between them (*p* < 0.05). Under severe drought conditions, with the increase in irrigation, the effect of alleviating drought stress is more obvious. Furthermore, a significant interaction was observed between drought severity and irrigation level, indicating that both factors coordinately regulate the germination attributes of foxtail millet seeds. These findings demonstrate that optimal irrigation management is crucial for enhancing germination quality.

In conclusion, appropriate irrigation optimizes the seed germination environment, effectively alleviates drought stress, promotes the coordinated growth of roots and shoots, and ultimately improves germination quality. Specifically, W3 is optimal under mild and moderate drought, while W4 performs best under severe drought conditions.

### 2.2. Transcriptomic Responses of Foxtail Millet Seeds to Irrigating-Sowing During Germination

**Figure 4:**
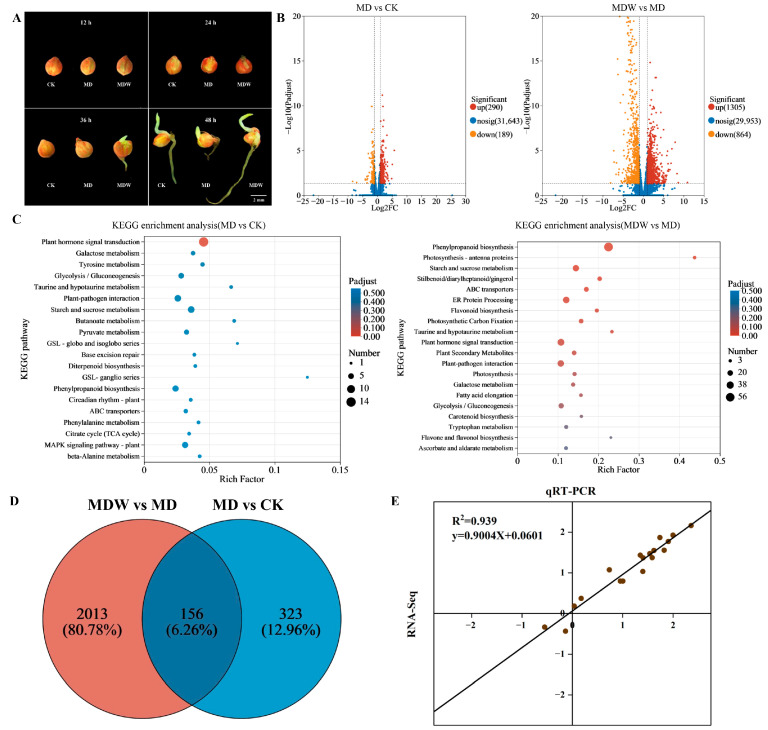
Transcriptional reprogramming induced by irrigating-sowing. (**A**) Changes in seeds per 12 h under CK, MD and MDW. (**B**) Volcano maps showing differentially expressed genes (DEGs) in different comparisons. (**C**) KEGG pathway enrichment analysis under different comparisons; the color scale is expressed as a significant level. (**D**) Venn diagram shows the overlapping DEGs in different comparisons. (**E**) Validation of transcriptome data by qRT-PCR.

To further characterize the transcriptional and metabolic responses associated with seeds germinating faster under irrigating-sowing, transcriptome sequencing was performed on foxtail millet seeds germinated for 48 h under three treatments: moderate drought with W0 (MD, 0 m^3^·hm^−2^), moderate drought with W3 (MDW, 13.5 m^3^·hm^−2^), and CK (field capacity 70% ± 5%). Morphological changes in seeds every 12 h under these treatments are shown in Figure 4A. In the comparisons of MD vs. CK and MDW vs. MD, 479 and 2169 differentially expressed genes (DEGs; |log_2_ fold change| > 1) were identified, respectively. Among them, 290 and 1305 genes were upregulated, and 189 and 864 were downregulated (Figure 4B). The Venn diagram illustrates the overlap of DEGs between the two comparisons (Figure 4D). KEGG pathway enrichment analysis revealed that these DEGs were mainly enriched in pathways related to plant hormone signal transduction, glycolysis, starch and sucrose metabolism, phenylpropanoid biosynthesis, and flavonoid biosynthesis (Figure 4C). To verify the reliability of the transcriptomic data, six DEGs were randomly selected for qRT-PCR analysis, covering key pathways identified in the study, including carbohydrate metabolism, secondary metabolism, and hormone signaling pathways, to ensure the representativeness of the validation. The results showed a high correlation between RNA-Seq and qRT-PCR data, confirming the accuracy of the transcriptome analysis (Figure 4E).

### 2.3. Carbohydrate and Energy Metabolism Responses to Irrigating-Sowing in Foxtail Millet

**Figure 5:**
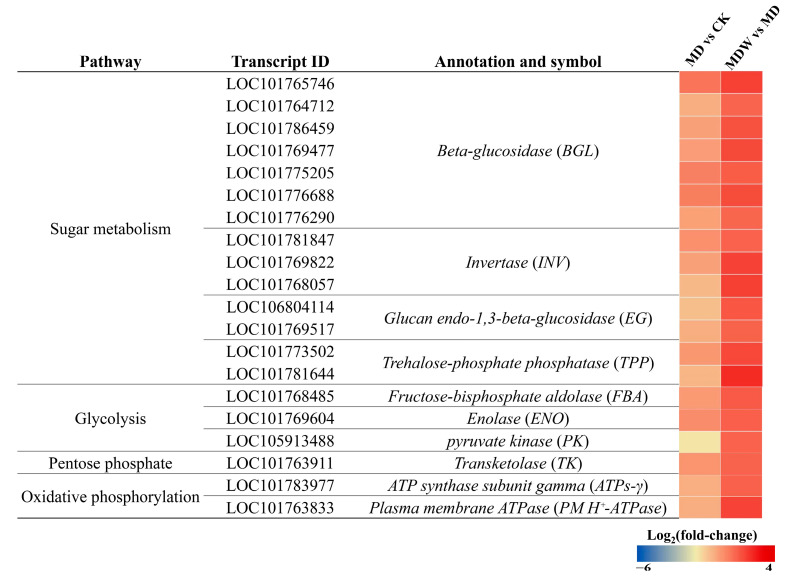
Changes in carbohydrate and energy metabolism under irrigating-sowing. The scale represents log_2_ (fold-change).

**Figure 6:**
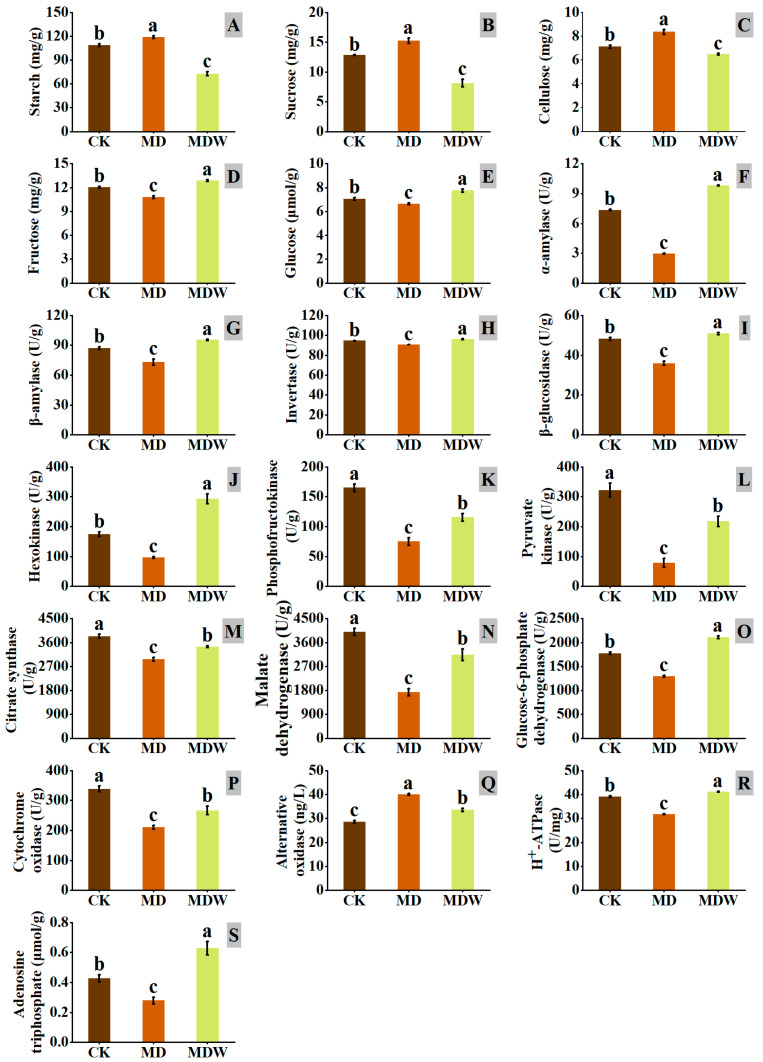
The changes in enzymes and substances related to carbohydrate and energy metabolism under Irrigating-sowing. (**A**) starch, (**B**) sucrose, (**C**) cellulose, (**D**) fructose, (**E**) glucose, (**F**) α-amylase (AMY), (**G**) β-amylase (BMY), (**H**) invertase (INV), (**I**) β-glucosidase (BGL), (**J**) hexokinase (HK), (**K**) phosphofructokinase (PFK), (**L**) pyruvate kinase (PK), (**M**) citrate synthase (CS), (**N**) malate dehydrogenase (MDH), (**O**) glucose-6-phosphate dehydrogenase (G6PDH), (**P**) cytochrome oxidase (CCO), (**Q**) alternative oxidase (AOX), (**R**) plasma membrane H^+^-ATPase (PM-H^+^-ATPase), and (**S**) ATP. The error bars represent the standard error of the mean. Different lowercase letters showed significant differences between different treatments (*p* < 0.05).

Under MDW treatment, carbohydrate metabolism and energy generation pathways changed significantly during foxtail millet seed germination. First, the mobilization of stored carbon sources was significantly enhanced. Compared to MD, MDW significantly increased the activities of AMY and BMY by 231.48% and 30.65%, respectively, while starch content decreased by 38.83%. Additionally, the transcript levels of genes encoding INV and BGL were higher under the MDW treatment (Figure 5), accompanied by increases of 6.03% and 41.64% in their corresponding enzyme activities, and decreases of 46.80% and 22.58% in sucrose and cellulose contents, respectively (Figure 6B,C). Furthermore, MDW significantly upregulated the expression of *EG* and *TPP* genes involved in UDP-glucose degradation and trehalose synthesis (Figure 5), and the contents of fructose and glucose increased by 19.53% and 16.93%, respectively, compared to MD (Figure 6D,E), indicating enhanced accumulation of soluble sugars, providing sufficient substrates for downstream energy metabolism.

In the glycolysis pathway, MDW significantly upregulated the expression of key enzymes such as *FBA*, *ENO*, and *PK* (Figure 5), and the corresponding enzyme activities were significantly enhanced. PK, PFK, and HK activities increased by 177.78%, 54.00%, and 203.85%, respectively (Figure 6J,K,L), leading to a significant increase in glycolytic flux and enhanced pyruvate production.

In the tricarboxylic acid cycle, MDW significantly increased the activity of CS and MDH by 16.04% and 81.82%, respectively, compared to MD (Figure 6M,N). This resulted in an accelerated rate of pyruvate oxidation and increased generation of NADH and other electron donors, providing sufficient substrates for subsequent oxidative phosphorylation.

In the pentose phosphate pathway, MDW significantly upregulated the expression of the *TK* gene (Figure 5), and the activity of G6PDH increased by 63.02% (Figure 6O), reflecting an increased rate of NADPH production, which helps maintain cellular redox balance.

During oxidative phosphorylation, MDW significantly increased the expression of *ATPs-γ* and *PMH^+^-ATPase* genes (Figure 5), leading to significant increases in enzyme activities, with cytochrome c oxidase (CCO) and PMH^+^-ATPase activities increasing by 26.40% and 29.82%, respectively (Figure 6P,R). In contrast, the activity of AOX decreased by 15.93% (Figure 6Q), indicating that more electrons were directed toward high-efficiency synthetic pathways. Finally, ATP content increased by 124.98% compared to MD (Figure 6S), demonstrating that MDW effectively enhanced oxidative phosphorylation efficiency and ATP production capacity.

### 2.4. Secondary Metabolism Responses to Irrigating-Sowing in Foxtail Millet

**Figure 7:**
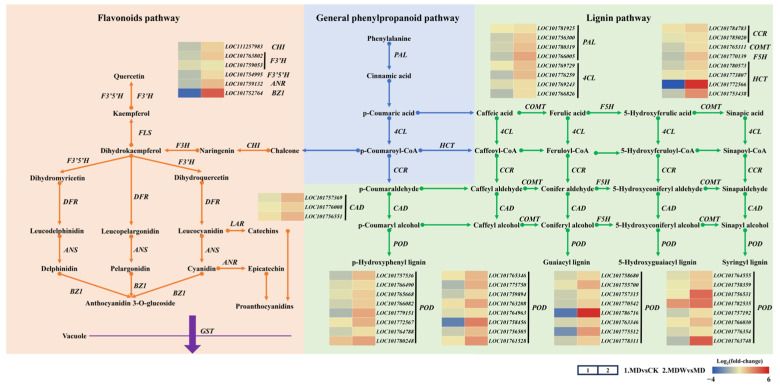
Changes in secondary metabolism under irrigating-sowing. The scale represents log_2_ (fold-change).

**Figure 8:**
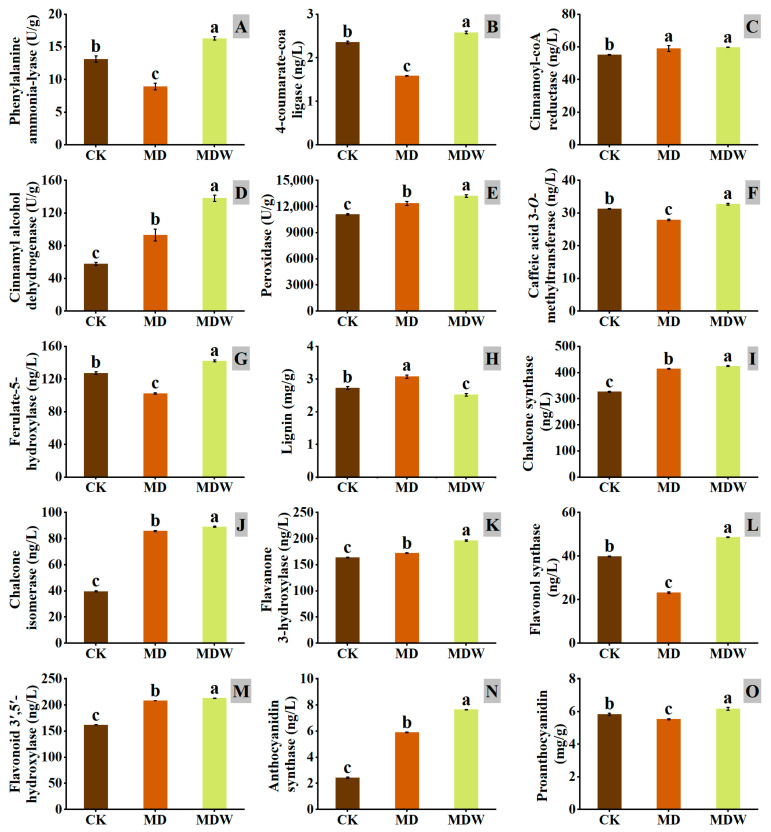
The changes in enzymes and secondary metabolites under irrigating-sowing. (**A**) phenylalanine ammonia lyase (PAL), (**B**) 4-coumarate coenzyme-A-ligase (4CL), (**C**) cinnamoyl-CoA reductase (CCR), (**D**) cinnamyl alcohol dehydrogenase (CAD), (**E**) peroxidase (POD), (**F**) caffeic acid 3-O-methyltransferase (COMT), (**G**) ferulic acid-5-hydroxylase (F5H), (**H**) lignin, (**I**) chalcone synthase (CHS), (**J**) chalcone isomerase (CHI), (**K**) flavanone 3-hydroxylase (F3H), (**L**) flavonol synthase (FLS), (**M**) flavonoid 3′,5′-hydroxylase (F3′5′H), (**N**) anthocyanin synthase (ANS), and (**O**) proanthocyanidin. The error bars represent the standard error of the mean. Different lowercase letters showed significant differences between different treatments *(p* < 0.05).

Under drought stress, the expression of genes involved in phenylpropanoid metabolism (*PAL*, *4CL*) was significantly downregulated, but irrigation reversed this change (Figure 7). Specifically, PAL and 4CL enzyme activities increased by 82.58% and 63.00%, respectively, under MDW compared to MD (Figure 8A,B).

The transcript abundance of *CCR*, a key gene in lignin biosynthesis, was higher under MDW than MD (Figure 7), corresponding, a 1.36% increase in CCR enzyme activity under MDW (Figure 8C). Drought stress downregulated the expression of other lignin biosynthesis genes (*COMT*, *F5H*, *HCT*, *POD*), but irrigation reversed this effect. MDW significantly increased COMT, F5H, and POD enzyme activities by 17.20%, 39.04%, and 6.82%, respectively (Figure 8E–G). Additionally, the transcript abundance of *CAD* was significantly higher under MDW, resulting in a 48.28% increase in *CAD* enzyme activity (Figure 8D). However, lignin content in MD was 21.89% higher than in MDW (Figure 8H).

In flavonoid biosynthesis-related genes (*CHI*, *F3H*, *F3′5′H*), irrigation significantly upregulated their expression, with transcript abundance being significantly higher under MDW than MD. Furthermore, the enzyme activities of CHS, CHI, F3H, F3′5′H, and FLS increased by 2.53%, 3.88%, 14.07%, 2.24%, and 109.44%, respectively, under MDW compared to MD (Figure 8I–M). Additionally, the transcript abundance of *ANR* and *BZ1*, involved in proanthocyanidin biosynthesis, was also significantly increased under MDW, correlating with a 29.82% increase in ANS activity. As a result, proanthocyanidin content increased by 11.46% in MDW compared to MD.

In summary, irrigation-sowing alleviated drought stress and reprogrammed phenylpropanoid metabolism, leading to enhanced activity of key enzymes in lignin biosynthesis and increased accumulation of flavonoids and proanthocyanidins. These changes are likely related to the alleviation of drought stress and the enhancement of seed germination potential, further confirming irrigation’s role in regulating plant secondary metabolism.

### 2.5. Changes in Plant Hormone Content and Signaling Pathways Under Irrigating-Sowing

**Figure 9:**
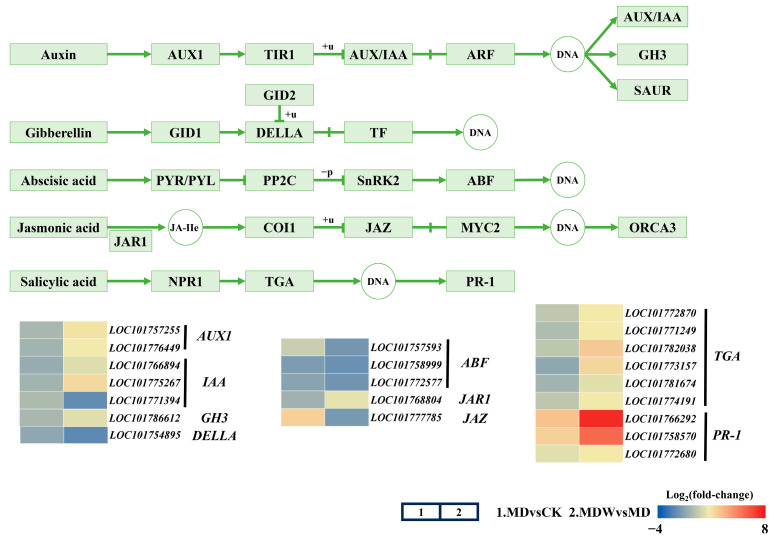
Changes in hormone signaling under irrigating-sowing. The scale represents log_2_ (fold-change).

KEGG pathway analysis showed that MDW upregulated the expression of *AUX1*, *IAA*, and *GH3* in the IAA pathway (Figure 9), enhancing auxin signaling, which supports cell elongation and polar growth. In the GA pathway, *DELLA* expression was downregulated, promoting GA activity. In the ABA pathway, *ABF* expression was reduced, indicating inhibition of ABA signaling and promoting germination. In the JA pathway, MDW reversed the downregulation of *JAR1* and reduced the expression of *JAZ*, activating JA signaling, which may regulate defense mechanisms. In the SA pathway, MDW enhanced *TGA* and *PR-1* expression, suggesting irrigation induces stronger defense mechanisms to support germination.

**Figure 10:**
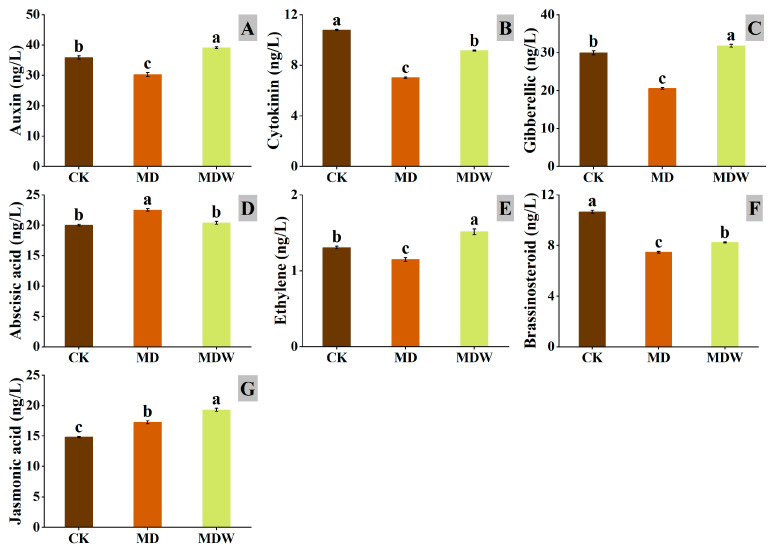
The changes in endogenous hormone contents under irrigating-sowing. (**A**) auxin (IAA), (**B**) cytokinin (CTK), (**C**) gibberellin (GA), (**D**) abscisic acid (ABA), (**E**) ethylene (ETH), (**F**) brassinolide (BR), and (**G**) jasmonic acid (JA). The error bars represent the standard error of the mean. Different lowercase letters showed significant differences between different treatments (*p* < 0.05).

Under MDW, several plant hormones in foxtail millet seeds significantly changed. Compared to MD, IAA, CTK, GA, ETH, BR, JA increased by 29.09%, 30.58%, 54.70%, 31.83%, 10.35%, and 11.85%, respectively, while ABA decreased by 9.35% (Figure 10A–G). These results indicate that irrigating-sowing is associated with altered hormone homeostasis that is consistent with a germination-favorable environment.

In summary, MDW reshapes endogenous hormonal homeostasis by activating germination-promoting pathways (IAA, GA, CTK, JA) and inhibiting ABA signaling, establishing a favorable environment for seed germination. Furthermore, the activation of JA and SA pathways enhances early defense responses, contributing to efficient germination under drought stress.

## 3. Discussion

### 3.1. Irrigating-Sowing Enhances Foxtail Millet Seed Germination Quality by Promoting Coordinated Root and Shoot Growth

Drought is a major abiotic stress that limits crop production, severely affecting plant metabolism and growth [39]. The seed germination to seedling emergence stage is the most water-sensitive period for crops [40]. This study systematically investigated the physiological and molecular responses of foxtail millet (Jingu 21) seeds to irrigating-sowing under drought conditions. The results showed that under mild and moderate drought conditions, pre-sowing irrigation of 13.5 m^3^/hm^2^ significantly improved germination energy, germination index, and vigor index. Under severe drought, irrigation at 18 m^3^/hm^2^ also alleviated the effects, indicating that appropriate irrigation improves soil moisture conditions, enhancing germination and seedling quality under dryland conditions.

Further analysis revealed that irrigating-sowing significantly promoted seed water absorption and metabolic activation, restoring both root and shoot lengths, with a more significant response in root growth. This indicates that under drought, roots exhibit prioritized growth to enhance water acquisition. This finding aligns with Voothuluru et al. [41], who reported that plants often maintain or increase root growth under water stress to improve water use efficiency. Additionally, Yan et al. [42] observed that soil moisture in the 0–20 cm layer directly impacts foxtail millet seedling emergence. Inadequate moisture prevents normal seed imbibition and germination. Our study showed that irrigation enhanced moisture in the sowing row, promoting rapid seed imbibition and successful germination. This demonstrates the practical field applicability of this method.

In conclusion, irrigating-sowing significantly alleviates drought-induced inhibition of seed germination, promotes coordinated root and shoot growth, and ultimately improves germination quality. This conclusion is consistent with the findings of Bayu et al. [43] and Queiroz et al. [13], who observed that a decline in water potential significantly suppresses germination, and further supports the practical effectiveness of pre-sowing irrigation in reversing drought’s adverse effects.

### 3.2. Irrigating-Sowing Enhances Carbohydrate Mobilization and Energy Metabolism During Seed Germination Under Drought Stress

Irrigation-sowing significantly promoted the degradation and utilization of stored carbohydrates such as starch and sucrose. During germination, starch serves as the main energy source, and its breakdown relies on AMY and BMY activities [17,44], which provide energy for cell division and elongation [45]. In this study, irrigation-sowing increased amylase activity, reduced starch content, and raised glucose and fructose levels. These findings contrast with Bai et al. [46], who reported reduced amylase activity and energy limitation under drought, indicating that irrigation-sowing alleviates drought-induced energy deficiency. Similarly, Song et al. [12] found that enhancing reducing sugar content and amylase activity mitigated drought stress in barley germination. Irrigation-sowing also enhanced INV and BGL activities, accelerating sucrose and cellulose degradation and increasing soluble sugars that act as osmolytes and respiratory substrates [47,48].

Moreover, energy metabolism was activated through higher activities of glycolytic enzymes including HK, PFK, and PK, which accelerated pyruvate formation and ATP production. This agrees with Li et al. [49] and Guo et al. [50], who reported that glycolytic enzymes maintain energy supply under drought conditions. Our results further demonstrate that irrigation-sowing not only sustains but also enhances the activities of these key enzymes, thereby improving the overall efficiency of energy metabolism and supporting rapid seed germination under water-limited conditions. In the tricarboxylic acid cycle, CS and MDH activities increased, enhancing organic acid metabolism and NADH production for the respiratory chain. CCO and plasma membrane H^+^-ATPase activities were upregulated, while AOX decreased, indicating more efficient electron flow toward ATP synthesis. These results contrast with Analin et al. [51] and Varshikar and Tan [52], who observed impaired electron transport and energy limitation under drought, highlighting the mitigating effect of irrigation-sowing. Additionally, G6PDH activity increased, enhancing PPP flux and NADPH production to maintain redox balance [19]. In summary, irrigation-sowing coordinates glycolysis, the TCA cycle, and the PPP to ensure sufficient and efficient energy supply for seed germination.

### 3.3. Irrigation-Sowing Regulates Secondary Metabolism to Enhance Stress Adaptation

Irrigation-sowing strengthened the phenylpropanoid pathway and its branches. Under MD, the expression of *PAL* and *4CL* decreased, while lignin content increased, indicating that foxtail millet reinforced cell walls through rapid lignin deposition to cope with stress. However, excessive lignification may restrict cell expansion and hinder germination [21,22]. With irrigation-sowing, lignin content declined, suggesting that carbon flux was redirected from lignin biosynthesis to other phenylpropanoid branches. The upregulation of *PAL*, *4CL*, *CCR*, and *CAD* indicated activation of the phenylpropanoid pathway, whereas increased expression of *CHS*, *CHI*, *F3H*, *FLS*, and *ANS* facilitated the accumulation of flavonoids, anthocyanins, and proanthocyanidins. These findings are consistent with Vogt [20], who emphasized the central role of the phenylpropanoid pathway in plant stress responses, and contrast with Yu et al. [53], who observed that PEG-induced drought suppressed phenylpropanoid metabolism and caused the accumulation of cinnamic acid derivatives that inhibited germination. The present results further demonstrate that irrigation-sowing restores phenylpropanoid activity and promotes flavonoid biosynthesis, thereby enhancing antioxidant defense while reducing the accumulation of potentially inhibitory metabolites, ultimately creating a more favorable metabolic environment for seed germination. Similarly, Bellaloui et al. [54] reported that phenylpropanoid-derived metabolites play key roles in drought tolerance in soybean, and our findings extend this understanding to the germination stage of foxtail millet under water-deficit conditions.

### 3.4. Irrigation-Sowing Reconfigures Endogenous Hormone Networks to Promote Germination

Irrigation-sowing significantly changed endogenous hormone content and signaling pathway activity. Drought typically induces ABA accumulation by enhancing its biosynthesis and suppressing its degradation, thereby inhibiting germination [24,55]. In this study, irrigation-sowing reduced ABA levels and the expression of its downstream signaling factor *ABF*, while increasing germination-promoting hormones such as IAA, GA, CTK, BR, and ETH. The GA signaling repressor *DELLA* was downregulated, facilitating GA-mediated growth. In the IAA pathway, the upregulation of *AUX1*, *IAA*, and *GH3* indicated enhanced auxin signaling, promoting cell elongation. Elevated CTK levels likely acted synergistically with GA to stimulate cell division and mesocotyl growth [34]. Moreover, irrigation-sowing enhanced JA and SA signaling, as evidenced by *JAZ* downregulation and *PR1* upregulation, suggesting strengthened defense preparedness during early germination [28]. These results indicate that irrigation-sowing reestablishes a hormonal balance favorable for germination by reducing the ABA/GA ratio and elevating IAA, CTK, and JA levels. This finding aligns with the model proposed by Sun and Gubler [25], in which the ABA/GA balance determines seed dormancy and germination.

It should be noted that the transcriptomic analysis was conducted only in 2024, and therefore the gene expression data reflect the responses of foxtail millet to irrigating-sowing under that specific drought environment. Although field experiments conducted in both 2023 and 2024 exhibited consistent physiological and morphological patterns (Supplementary Figures S1 and S2), the transcriptomic results should be interpreted as conditional rather than universal. Moreover, the RNA-seq data were generated from bulk seed samples collected 48 h after sowing, representing the average transcriptional state across multiple cell types and germination stages. Because seed germination is a highly coordinated multicellular process, variations in transcript abundance may partly reflect shifts in the proportion or activation of different cell types rather than direct transcriptional regulation. Consequently, the present findings reveal overall molecular trends associated with irrigating-sowing rather than cell-specific regulatory events. Future studies incorporating multi-year, multi-environment, and cell-type–resolved transcriptomic analyses will be required to confirm and refine these results.

**Figure 11:**
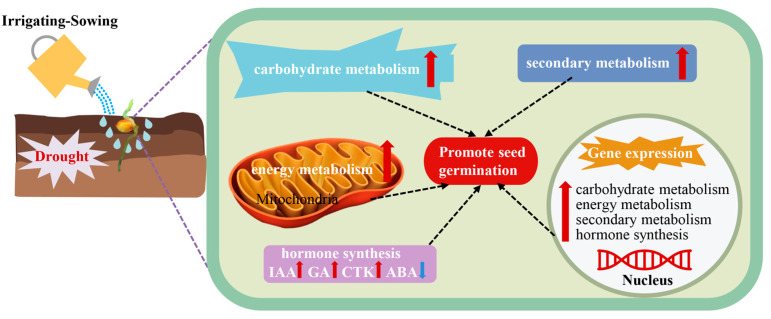
Irrigating-sowing promotes foxtail millet seed germination under drought stress, as reflected by coordinated enhancements in carbohydrate mobilization, energy metabolism, secondary metabolite synthesis, and hormone balance. Red arrows indicate increases, blue arrows indicate decreases, and dashed lines represent associations among processes.

In summary, irrigation-sowing promotes foxtail millet germination under drought by integrating multiple regulatory processes. It improves soil moisture and root growth, activates carbohydrate mobilization and energy metabolism, strengthens phenylpropanoid and flavonoid pathways, and reconfigures hormonal networks (Figure 11). The findings provide new insights into water-mediated germination regulation and offer practical guidance for dryland farming. In arid regions such as the Loess Plateau, irrigation-sowing can substantially improve seedling emergence and establishment, thereby supporting stable foxtail millet production.

## 4. Materials and Methods

### 4.1. Experimental Materials

In this experiment, high-quality representative foxtail millet variety Jingu 21 was selected as the research material, which was provided by the Institute of Economic Crops, Shanxi Agricultural University, Taigu, China.

### 4.2. Experimental Site

The experiment was conducted at the Experimental Station of Shanxi Agricultural University (112°55′ E, 37°42′ N, altitude 787.13 m) starting on 12 May 2024. The site is characterized by a semi-arid continental monsoon climate. The soil is classified as sandy loam, with the following properties: soil organic matter content 21.5–24.8 g·kg^−1^, total nitrogen 1.259–1.363 g·kg^−1^, total phosphorus 1.099–1.220 g·kg^−1^, total potassium 14.9–18.1 g·kg^−1^, available phosphorus 10.3–17.2 mg·kg^−1^, available potassium 177–245 mg·kg^−1^, and pH 8.43–8.51. The average daily temperature during the foxtail millet seedling stage was 22.91 °C, with a maximum of 29.24 °C and a minimum of 16.56 °C.

### 4.3. Experimental Design

The experiment adopted a two-factor split-plot design. The main factor was the soil drought level, categorized according to the agricultural drought classification standards (GB/T 32136-2015) set by the National Agricultural Meteorological Standardization Technical Committee (SAC/TC 539) [56]. Three drought levels were set: mild drought (60%±5% field water holding capacity), moderate drought (50 ± 5%), and severe drought (40 ± 5%). Soil moisture at 0–20 cm depth was measured to confirm that the soil reached severe drought levels before sowing in 2024. The soil bulk density was 1.24 g/cm^3^, and the maximum field water holding capacity was 25.69%. Based on these measurements, water supplementation was calculated to achieve light and moderate drought conditions (Table 1) [57]. The sub-factor was irrigation volume. Before sowing, water volumes of 0 m^3^/hm^2^, 4.5 m^3^/hm^2^, 9 m^3^/hm^2^, 13.5 m^3^/hm^2^, and 18 m^3^/hm^2^ were applied to the sowing furrows, labeled as W0, W1, W2, W3, and W4, with field capacity (70% ± 5%) as CK. The irrigation volumes were based on pre-experiment results from 2022 and 2023, as well as references from studies on hill-drop irrigation in foxtail millet [58,59]. Field row spacing was set at 50 cm, with seeds sown at a depth of 3 cm using a drill planting method. Each plot measured 19.25 m^2^ (5.5 m × 3.5 m) and contained 7 rows. The planting density for Jingu 21 was maintained at 375,000–450,000 plants per hectare. To prevent water infiltration between plots, each plot was surrounded by a 0.5 m partition, and a movable rain shelter was installed to block rain during rainfall while maintaining natural light during other times. The nitrogen, phosphorus, and potassium fertilizer application rate was consistent across all treatments, using nitrate phosphate-potassium compound fertilizer (Tianji Coal Chemical Group Co., Ltd., Tianjin, China, N-P_2_O_5_-K_2_O, 22-8-10) at a rate of 600 kg/hm^2^ (containing 132 kg/hm^2^ N, 48 kg/hm^2^ P_2_O_5_, and 60 kg/hm^2^ K_2_O) as a single basal dose applied to the 0–20 cm soil layer, with no additional fertilizers applied. Other field management practices were consistent with local farmer habits.

### 4.4. Determination of Seed Germination Characteristics

According to the germination phenotype of foxtail millet, representative seeds from each treatment were photographed and observed for morphological differences. The lengths of roots and shoots were measured with a ruler on Days 2, 4, 6, and 8 after sowing, and the number of germinated seeds was recorded daily. On Day 6, the lengths of the embryo root and coleoptile were measured for germinated seeds. The germination rates on Days 2, 4, 6, and 8 were denoted as *nd*_2_, *nd*_4_, *nd*_6_, and *nd*_8_, respectively. Germination parameters were calculated following the method of Bukhari et al. [60].

The promptness index (PI) was calculated as:

PI = 1.00 × nd_2_ + 0.75 × nd_4_ + 0.50 × nd_6_ + 0.25 × nd_8_ (1)

The promptness index of drought resistance (PDI) was determined as:

(2) $$\mathrm{PDI}\;=\;\frac{{PI}_{treatment}}{{PI}_{control}}\times \;100\%$$

The vigor index (VI) was calculated as:

VI = GR × S_x_ (3)

where *S_x_* is the average shoot length on Day 6.

The vigor index of drought resistance (VDI) was calculated as:

(4) $$\mathrm{VDI}\;=\;\frac{{VI}_{treatment}}{{VI}_{control}}\times \;100\%$$

The germination energy (GE) was calculated as:

(5) $$\mathrm{GE}\;=\;\frac{N_{4d}}{N_{total}}\times \;100\%$$

where N_4d_ is the number of seeds germinated by Day 4, and N_total_ is the total number of tested seeds.

The germination index (GI) was calculated as:

(6) $$GI=\sum \frac{Gt}{Dt}$$

where *G_t_* is the number of seeds germinated on day *t*, and *D_t_* is the corresponding day of germination.

### 4.5. Determination of Carbohydrate Metabolism

Samples for all biochemical assays, hormone measurements, and transcriptome sequencing were collected 48 h after sowing. The seed coat and pericarps were removed to obtain embryos; for germinated seeds, the embryonic root and coleoptile were separated and immediately stored at −80 °C. Starch content was determined using a starch assay kit (STA20, Sigma-Aldrich, St. Louis, MO, USA) following Dong et al. [61]. Approximately 0.1 g of sample was homogenized in 0.8 mL of distilled water. The activities of α-amylase (AMY) and β-amylase (BMY) were quantified spectrophotometrically at 540 nm using commercial kits (Solarbio, Beijing, China) according to Zhang et al. [62,63] with a UV-2600 spectrophotometer (Shimadzu, Kyoto, Japan).

The concentrations of sucrose, fructose, and glucose were analyzed by high-performance liquid chromatography (HPLC, Waters 2695 series, Waters Corp., Milford, MA, USA) following Jia et al. [64] and Liu et al. [65]. Invertase (INV) activity was determined as described by Shahid et al. [66].

Cellulose content was measured using the anthrone colorimetric method [67]. Briefly, 0.2 g of sample was digested with sulfuric acid for 30 min, diluted to 100 mL, and reacted with an anthrone–sulfuric acid reagent. After incubation for 12 min, absorbance was recorded at 620 nm.

The activity of β-glucosidase (BGL) was determined with a commercial kit (Solarbio, Beijing, China). About 0.2 g of sample was homogenized in 1 mL of extraction buffer on ice and centrifuged at 15,000× *g* for 20 min at 4 °C. The supernatant was used for enzymatic assays according to the manufacturer’s protocol, and absorbance was read at 400 nm.

### 4.6. Determination of Glycolytic Enzyme Activities

The activities of hexokinase (HK), phosphofructokinase (PFK), and pyruvate kinase (PK) were determined using ELISA kits (Solarbio, Beijing, China) according to the manufacturer’s protocols. Enzyme activities were expressed as μmol·min^−1^·g^−1^ fresh weight [68].

The activities of cytochrome c oxidase (CCO) and glucose-6-phosphate dehydrogenase (G6PDH) were measured using commercial kits (BC0940 and BC0260, Solarbio, Beijing, China). For CCO, mitochondria were isolated by differential centrifugation, and absorbance changes were monitored at 550 nm. G6PDH activity was determined from supernatant extracts by measuring NADPH production at 340 nm. The content of alternative oxidase (AOX) was determined using ELISA with absorbance read at 450 nm.

Malate dehydrogenase (MDH) and citrate synthase (CS) activities were assayed following Kumar et al. [69] and Terrier et al. [70], respectively. The MDH assay monitored absorbance at 340 nm, while the CS assay monitored absorbance at 412 nm. Plasma membrane H^+^-ATPase activity was analyzed according to Li et al. [71] based on inorganic phosphorus release at 660 nm. ATP content was quantified using an ATP assay kit (Solarbio, Beijing, China).

### 4.7. Determination of Phenylpropanoid Enzymes and Lignin

The activities of phenylalanine ammonia-lyase (PAL) and 4-coumarate-CoA ligase (4CL) were measured following Li et al. [72,73] and Wei et al. [74]. One unit of PAL or 4CL activity was defined as a 0.01 absorbance increase at 290 or 333 nm, respectively.

Peroxidase (POD) activity was assayed according to Phimchan et al. [75] using guaiacol and H_2_O_2_ as substrates, and absorbance was recorded at 470 nm.

The contents of ferulate-5-hydroxylase (F5H), cinnamoyl-CoA reductase (CCR), and caffeic acid O-methyltransferase (COMT) were determined using ELISA kits (Solarbio, Beijing, China) at 450 nm, while cinnamyl alcohol dehydrogenase (CAD) activity was assayed spectrophotometrically at 340 nm. Enzyme activities were expressed as U·g^−1^ fresh weight.

Lignin content was measured following Kim et al. [76]. Samples were sequentially extracted with ethanol and dimethyl sulfoxide, and the final residue was dissolved in NaOH for spectrophotometric determination at 280 nm.

### 4.8. Determination of Flavonoid and Anthocyanin Pathway Enzyme Activities and Metabolites

Chalcone isomerase (CHI) activity was determined following Lam et al. [77]. Frozen samples were ground in liquid nitrogen, extracted with 0.05 M HEPES–NaOH buffer (pH 7.5), and centrifuged. The reaction mixture contained crude protein, buffer, ethanol, and naringenin chalcone, and absorbance was monitored at 390 nm.

The contents of chalcone synthase (CHS), flavanone 3-hydroxylase (F3H), flavonol synthase (FLS), flavonoid 3′5′-hydroxylase (F3′5′H), and anthocyanidin synthase (ANS) were quantified using ELISA kits (Kebo Biotechnology, China) with absorbance read at 450 nm.

Proanthocyanidins (PAs) were determined following Verardo et al. [78] using HPLC-FLD-ESI-MS after extraction with 80% ethyl acetate and filtration through a 0.2-μm PTFE membrane.

### 4.9. Determination of Plant Hormones

Plant hormones were extracted and quantified following Wang et al. [68]. Take 0.5 g of the sample, grind it in an ice bath, add 5 mL of 80% (*v*/*v*) methanol, centrifuge at 8000× *g* for 10 min at 4 °C, and collect the supernatant. The concentrations of auxin (IAA), abscisic acid (ABA), cytokinin (CTK), gibberellin (GA), brassinosteroids (BRs), and jasmonic acid (JA) were determined using enzyme-linked immunosorbent assay (ELISA) kits according to the manufacturer’s instructions.

### 4.10. RNA Extraction and RNA-Seq Assay

After 48 h of sowing, foxtail millet seeds were sampled and used for RNA-Seq. Total RNA was extracted from the sampled seeds using a TRIzol kit and submitted to Shanghai Majorbio Bio-Pharm Technology Co., Ltd. for sequencing. Quality control was performed with fastp (Version 0.23.4) [79] to obtain clean reads. The clean reads were aligned to the reference genome using HISAT2 (http://ccb.jhu.edu/software/hisat2/index.shtml) (accessed on 1 November 2025) [80]. The *Setaria italica* reference genome used was GCF_000263155.2_Setaria_italica_v2.0 (https://ftp.ncbi.nlm.nih.gov/genomes/all/GCF/000/263/155/GCF_000263155.2_Setaria_italica_v2.0/) (accessed on 1 November 2025). Quantification was conducted with RSEM (Version 1.3.3) to obtain gene-level expression. Differentially expressed genes were identified using the DESeq2 R package (v1.42.0) with the criteria FDR < 0.05 and |log_2_FC| ≥ 1. KEGG annotation was performed using the KEGG database (https://www.genome.jp/kegg/) (accessed on 1 November 2025). KEGG enrichment analysis of significant DEGs was carried out with the Python SciPy package (v1.6.3) (https://scipy.org/install/) (accessed on 1 November 2025) using Fisher’s exact test. To control the false discovery rate, *p*-values were adjusted by the Benjamini–Hochberg (BH) method, and pathways with adjusted *p* < 0.05 were considered significantly enriched. Each treatment included three biological replicates. All RNA-Seq data have been deposited in the NCBI Sequence Read Archive (SRA) under BioProject accession number PRJNA1359106 (https://www.ncbi.nlm.nih.gov/bioproject/1359106) (accessed on 1 November 2025).

### 4.11. Quantitative Real-Time PCR Validation

First-strand cDNA was synthesized from total RNA using the Hifair^®^ III 1st Strand cDNA Synthesis SuperMix for qPCR (gDNA digester plus) kit. Quantitative real-time PCR (qRT-PCR) was performed with the Hieff^®^ qPCR SYBR Green Master Mix (Low Rox Plus) kit on a Bio-Rad CFX96 system (Bio-Rad, Hercules, CA, USA). The *Actin* gene was used as an internal reference, and relative expression levels were calculated using the 2^−ΔΔCt^ method [81]. Six DEGs were randomly selected for qRT-PCR to validate the RNA-seq results. The primer sequences used in this study are listed in Table 2.

### 4.12. Statistical Analysis

Data from three biological replicates are presented as the mean ± standard error (SE). A two-way analysis of variance (ANOVA) was conducted to evaluate the main effects of drought level, irrigation amount, and their interaction. Significant differences among treatment means were determined using Duncan’s multiple range test at *p* < 0.05. All statistical analyses were performed using SPSS Statistics 26.0 (IBM Corp., Armonk, NY, USA). Figures were generated with Origin 2021 (OriginLab, Northampton, MA, USA), and heatmaps were visualized using TBtools-II.

## 5. Conclusions

This study combined field experiments with physiological and transcriptomic analyses to characterize how foxtail millet seeds respond to irrigation-sowing under drought conditions. Irrigation-sowing improved soil moisture and facilitated coordinated root–shoot growth, contributing to better germination quality. Transcriptomic and physiological evidence indicated that irrigated seeds exhibited metabolic states associated with stronger glycolysis, the tricarboxylic acid cycle, and the pentose phosphate pathway, collectively supporting more active energy metabolism. Pathways related to phenylpropanoid and flavonoid metabolism also showed higher activity-associated signatures, accompanied by greater accumulation of antioxidant-related metabolites. In addition, irrigation was associated with a reduced the ABA/GA ratio and increased the relative contents of IAA, CTK, and GA, thereby reestablishing a hormonal balance favorable for germination initiation. In summary, irrigation-sowing functions as an upstream water-supply signal that reshapes metabolic activity and hormonal balance in ways associated with improved early development. This integrated response substantially improves seed germination and seedling establishment under drought, providing theoretical and practical guidance for improving emergence rate and crop stability in arid regions.

## Figures and Tables

**Table 1:** Soil water content (%) at 0–20 cm under different drought levels.

Years	Mild Drought (%)	Moderate Drought (%)	Severe Drought (%)	CK (%)
2024	16.14 ± 0.28	13.2 ± 0.73	9.31 ± 0.22	17.02 ± 0.32

**Table 2:** qRT-PCR primer sequence.

Gene-ID	Forward Primer Sequences (5′-3′)	Reverse Primer Sequences (5′-3′)
*LOC101748783*	TGGGTCAGGTGTAGTTTCTTG	GCATGTGTAGGGTAGTAGGTAAG
*LOC111257983*	GCGGCTGCGTGATTATATTG	AACACACGAGATGACCGATAG
*LOC101786459*	GTTTGGAGACAGGGTCAAGAA	GCATCTGCCTGGTGCTAATA
*LOC101781925*	TCACAAGCTCTTCTCCAACAC	GGGATAGAACTGAAAGCACTAAGA
*LOC101776259*	CCTGGGACAGCCATTACATTA	CAACCGGGATTTCTCCAATTTC
*LOC101754995*	CCGCCTCCCTCTAATTCTAAAC	CTTCTCTCGGTTGAGTGTTCTG
*ACTIN*	CGCATATGTGGCTCTTGACT	GGGCACCTAAATCTCTCTGC

## Data Availability

The data that support this study are available upon reasonable request from the corresponding author. The data are not publicly available due to privacy.

## Supplementary Materials

The following supporting information can be downloaded at: https://www.mdpi.com/article/10.3390/plants14233571/s1, Figure S1: Effects of irrigating-sowing on root and shoot growth of foxtail millet. Figure S2: The changes of germination energy (**A**), germination index (**B**), promptness index (**C**), vigor index (**D**), promptness index of drought resistance (**E**) and vigor index of drought resistance (**F**) of foxtail millet seeds under irrigating-sowing.
