# Supplementary material for: Physiological and Molecular Responses of Seed Germination to Irrigating-Sowing in Drought-Stressed Foxtail Millet (Setaria italica L.)
Source: Plants (Basel). 2025 Nov 22;14(23):3571. doi: 10.3390/plants14233571 (PMC12693747; doi:10.3390/plants14233571)
Supplement: Supplementary file 1 [file plants-14-03571-s001.zip › plants-3979021-supplementary.pdf]

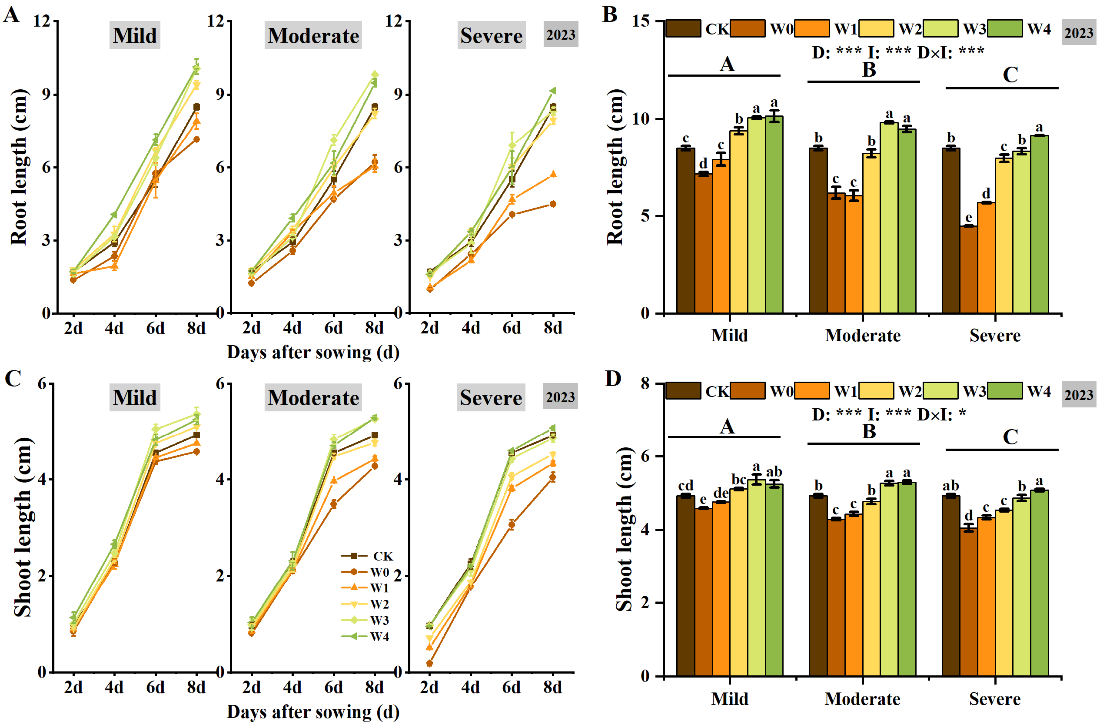

Supplementary Figure S1. Effects of irrigating-sowing on root and shoot growth of foxtail millet. (A, C) Changes in root (A) and shoot (C) length of foxtail millet seedlings at 2, 4, 6, and 8 days after sowing under three drought levels (mild, moderate, and severe) with five irrigation treatments (W0–W4). CK represents the well-watered control (70% ± 5% of field capacity). (B, D) Comparison of root (B) and shoot (D) length on the 8th day under different drought and irrigation treatments. Error bars represent the standard error of the mean (n = 3). Different uppercase letters indicate significant differences among drought levels ($p < 0.05$), and different lowercase letters indicate significant differences among irrigation treatments within the same drought level ($p < 0.05$). D, I, and D × I represent the main effects of drought, irrigation, and their interaction, respectively. *, **, and *** indicate significant differences at $p < 0.05$, $p < 0.01$, and $p < 0.001$, respectively.

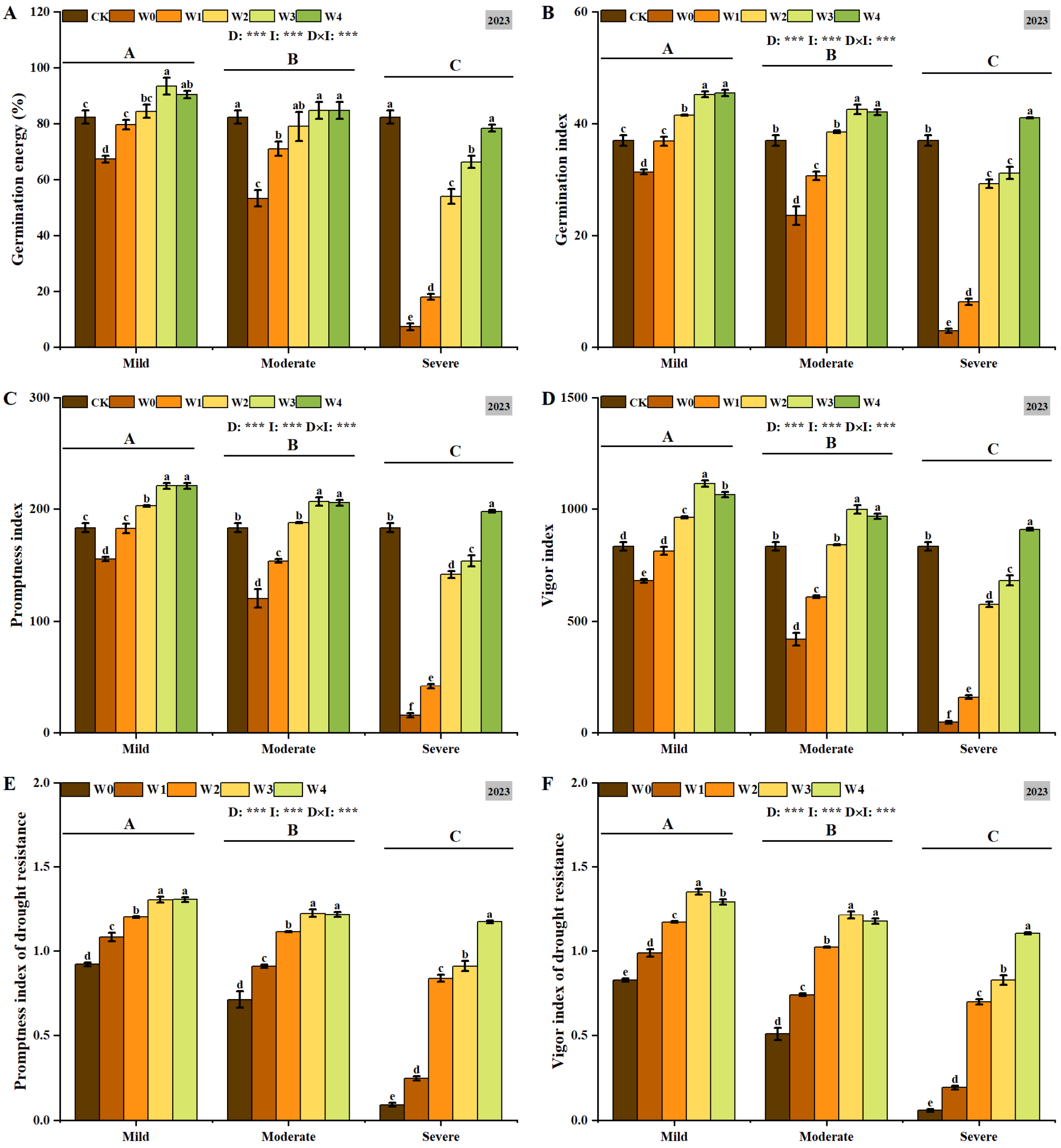

Supplementary Figure S2. The changes of germination energy (**A**), germination index (**B**), promptness index (**C**), vigor index (**D**), promptness index of drought resistance (**E**) and vigor index of drought resistance (**F**) of foxtail millet seeds under irrigating-sowing. Error bars represent the standard error of the mean (n = 3). Different uppercase letters indicate significant differences among drought levels (p < 0.05), and different lowercase letters indicate significant differences among irrigation treatments within the same drought level (p < 0.05). D, I, and D × I represent the main effects of drought, irrigation, and their interaction, respectively. *, **, and *** indicate significant differences at p < 0.05, p < 0.01, and p < 0.001, respectively.
